# Fast-track virtual reality software to facilitate 3-dimensional reconstruction in congenital heart disease

**DOI:** 10.1093/icvts/ivad087

**Published:** 2023-06-14

**Authors:** Francesco Bertelli, Francesca Raimondi, Charlotte Godard, Emma Bergonzoni, Claudia Cattapan, Elisa Gastino, Francesco Galliotto, Nathalie Boddaert, Mohamed El Beheiry, Jean-Baptiste Masson, Alvise Guariento, Vladimiro L Vida

**Affiliations:** Pediatric and Congenital Cardiac Surgery Unit, University of Padua, Italy; Pediatric Cardiology Unit, Meyer Hospital, University of Florence, Italy; Decision and Bayesian Computation, Computation Biology Department, CNRS, URS 3756, Neuroscience Department, CNRS UMR 3571, Institut Pasteur, Paris, France; Decision and Bayesian Computation, Computation Biology Department, CNRS, URS 3756, Neuroscience Department, CNRS UMR 3571, Institut Pasteur, Paris, France; Pediatric and Congenital Cardiac Surgery Unit, University of Padua, Italy; Pediatric and Congenital Cardiac Surgery Unit, University of Padua, Italy; Pediatric and Congenital Cardiac Surgery Unit, University of Padua, Italy; Pediatric and Congenital Cardiac Surgery Unit, University of Padua, Italy; Pediatric Radiology Unit, Hôpital Universitaire Necker-Enfants Malades, Université de Paris, France; Decision and Bayesian Computation, Computation Biology Department, CNRS, URS 3756, Neuroscience Department, CNRS UMR 3571, Institut Pasteur, Paris, France; Decision and Bayesian Computation, Computation Biology Department, CNRS, URS 3756, Neuroscience Department, CNRS UMR 3571, Institut Pasteur, Paris, France; Pediatric and Congenital Cardiac Surgery Unit, University of Padua, Italy; Pediatric and Congenital Cardiac Surgery Unit, University of Padua, Italy

**Keywords:** Congenital Heart Disease, 3D Reconstruction, Virtual Reality, DIVA software

## Abstract

**OBJECTIVES:**

Two limitations of the clinical use of 3-dimensional (3D) reconstruction and virtual reality systems are the relatively high cost and the amount of experience required to use hardware and software to effectively explore medical images. We have tried to simplify the process and validate a new tool developed for this purpose with a novel software package.

**METHODS:**

Five patients with right partial anomalous pulmonary venous return with adequate preoperative images acquired with magnetic resonance imaging were enrolled. Five volunteers with no previous experience in the field of 3D reconstruction were instructed to use the software after viewing a short video tutorial. Users were then asked to create a 3D model of each patient's heart using DIVA software. Their results were compared quantitatively and qualitatively with a benchmark reconstruction performed by an experienced user.

**RESULTS:**

All our participants recreated 3D models in a relatively short time, maintaining a good overall quality (average quality score ≥ 3 on a scale of 1–5). The overall trend of all the parameters analysed showed a statistical improvement between case 1 and case 5, as users became more and more experienced.

**CONCLUSIONS:**

DIVA is a simple software program that allows accurate 3D reconstruction in a relatively short time (“fast-track” virtual reality). In this study, we demonstrated the potential use of DIVA by inexperienced users, with a significant improvement in quality and time after a few cases were performed. Further studies are needed to confirm the potential application of this technology on a larger scale.

**Figure:**
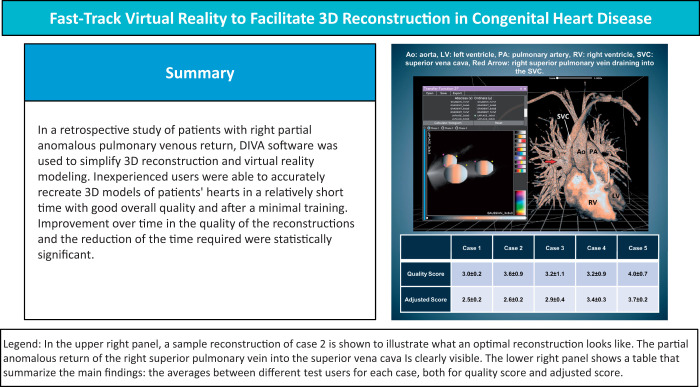

## INTRODUCTION

Computer-generated 3-dimensional (3D) reconstruction is emerging as a growing technology in the world of congenital heart disease (CHD). The benefits of virtual reality (VR) or 3D printed models have been clearly demonstrated, especially when dealing with complex anatomies or in the case of planning minimally invasive procedures [1]. Indeed, a deeper and more extensive understanding of the spatial relationship between the different anatomical structures allows a superior surgical approach, changing it entirely in some cases [2]. Nevertheless, further large-scale studies are required to demonstrate the potential for 3D reconstruction to reduce operative time or prevent complications in congenital heart surgery, as has already been achieved in other surgical fields [3–4].

Nevertheless, one limitation of the clinical use of these systems is the relatively high cost and the degree of expertise required to use the currently available software. In addition, the spread of these technologies has been limited by the lack of standardized approaches, long processing times and a lack of dynamic representations of the cardiac cycle. As this field develops, new options are becoming available with a potential simplification of the complexity of the platforms required to obtain virtual models.

The DIVA software (Data Integration and Visualization in Augmented and Virtual Environments, Institut Pasteur, Paris) is a new VR technology allowing fast and user-friendly 3D reconstruction of CHD [5]. We previously compared this software to standard 3D rendering techniques and concluded that DIVA was systematically consistent and faster [6]. In the present study, we analysed the use of this software by users with limited expertise to evaluate the potential for simplification of 3D reconstruction in CHD.

## MATERIALS AND METHODS

### Ethics statement

Patient data were completely anonymized so that neither informed consent nor institutional review board approval was required.

### Study design

A cohort of patients diagnosed with right partial anomalous pulmonary venous return (PAPVR) and associated superior sinus venous defect was selected from among all patients who underwent surgery at the University of Padua between January 2015 and December 2020. The exclusion criteria were the unavailability of medical imaging, imaging performed after surgical correction, any imaging techniques other than cardiac magnetic resonance imaging (MRI), and patients whose imaging was otherwise unsuitable for 3D reconstruction due to poor quality or lack of 3D acquisitions.

Cardiac MRI data from 5 patients were selected and used to build the reconstructed 3D models. Cases 1, 2, 3 and 5 were 8-, 31-, 26- and 4-year-old females, respectively. Case 4 was a 10-year-old boy.

We then recruited 5 participants with little or no expertise in 3D reconstruction, including 2 medical students and 3 cardiac surgery residents, all with little experience in the field of CHD. An expert user (FB) with years of experience in the field of 3D reconstruction of CHD and time spent with the DIVA software recorded a 40-min video tutorial for the participants. The participants were allowed to review the video as often as they wanted a few days before the actual reconstructions. For the tutorial, a sample case was selected for didactic purposes among the patients excluded from the study, to avoid any simple emulation among test users. This tutorial was designed to guide completely novice users through the complete pipeline of the reconstruction process. After this, we asked the participants to generate PAPVR VR models of the 5 patients using the DIVA software. The expert user also made 3D reconstructions of all the cases.

**Figure 1:**
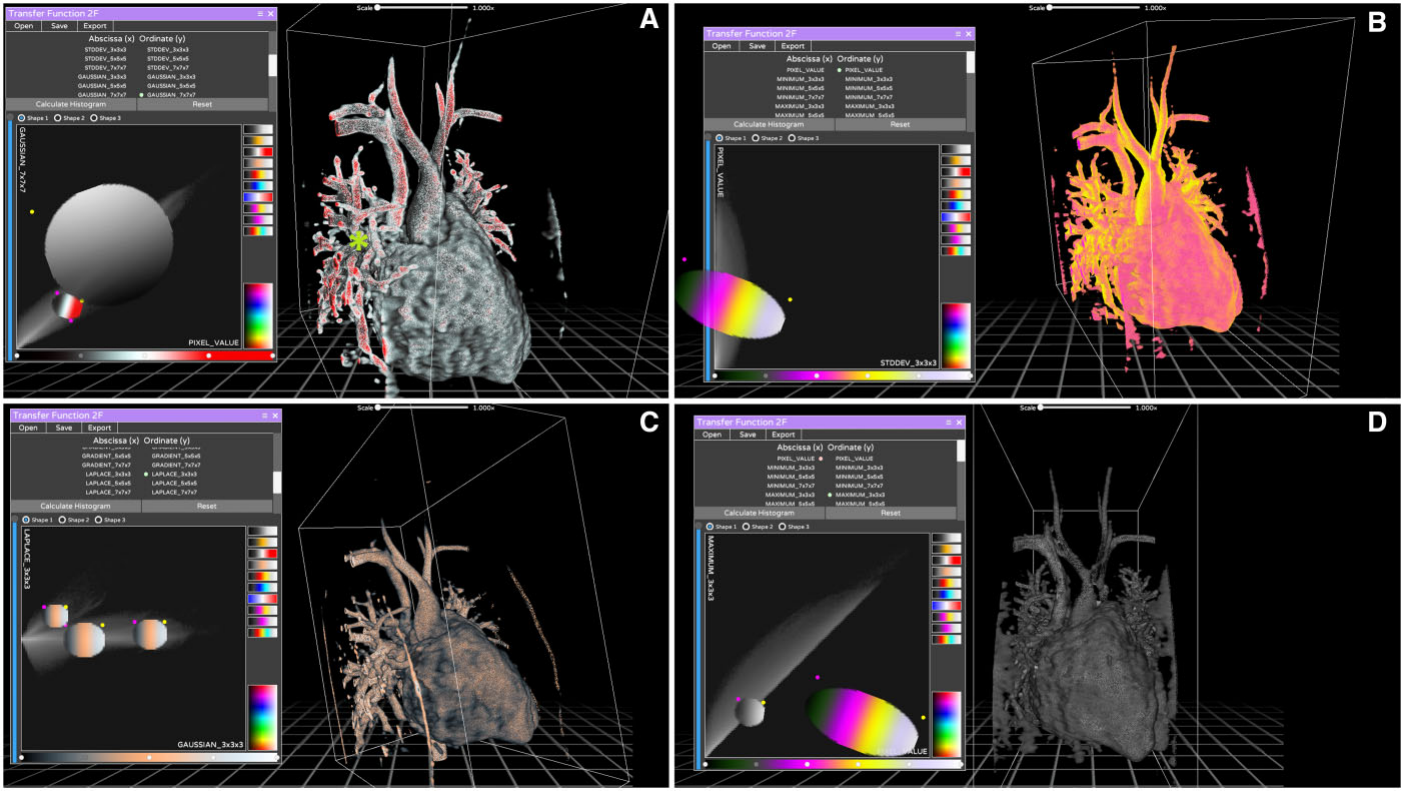
Reconstructions of case 1 generated by the participants. The diagnosis was partial anomalous pulmonary venous return of the right superior pulmonary vein into the superior vena cava well above the origin of the azygos vein. The right middle pulmonary vein drained into the junction between the superior vena cava and the right atrium. This defect was associated with a rather large sinus venous defect. Panel A shows the reference model created by the skilled user. A large percentage of voxels are selected using only 2 large circle filters, both greyscale and colour. The partial anomalous pulmonary venous return is clearly visible and distinguishable from other structures. A small green star shows the defect itself. Panel B shows a model with too much colour and no contrast. Panel C shows a reconstruction with quality comparable to the reference model. Panel D shows a complete lack of colour due to an improperly used filter.

**Figure 2:**
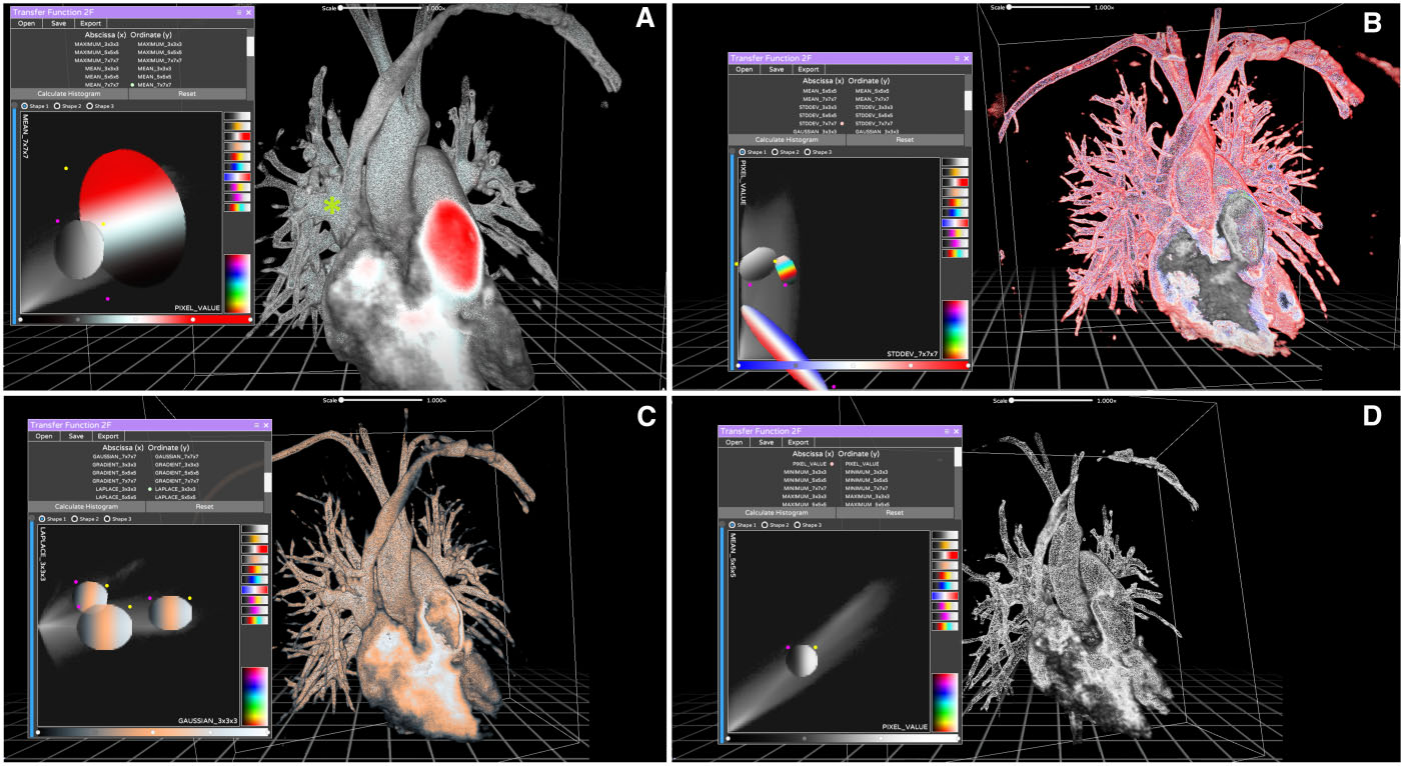
Reconstructions of case 2 generated by the participants. The diagnosis was partial anomalous pulmonary venous return of 2 separate right upper pulmonary veins in the superior vena cava. The right middle pulmonary vein drained into the junction between the superior vena cava and the right atrium, where there was a small sinus venous defect. Panel A shows the reference model created by the expert user. Again, a large percentage of voxels are selected using only 2 large circular filters, one chromatic and one greyscale. The partial anomalous pulmonary venous return is highly visible and distinguishable from other structures, and the inside of the model is also different from the outside (this can best be viewed using appropriate virtual reality equipment). A small green star shows the defect itself. Panel B shows a pattern with too much noise, misuse of colour and poor separation between venous and arterial structures. Panel C shows a reconstruction whose quality is comparable to that of the reference model, although without good colour separation. Panel D shows a complete lack of colour due to the use of a single greyscale filter. The model is also poorly defined and difficult to navigate using virtual reality equipment.

**Figure 3:**
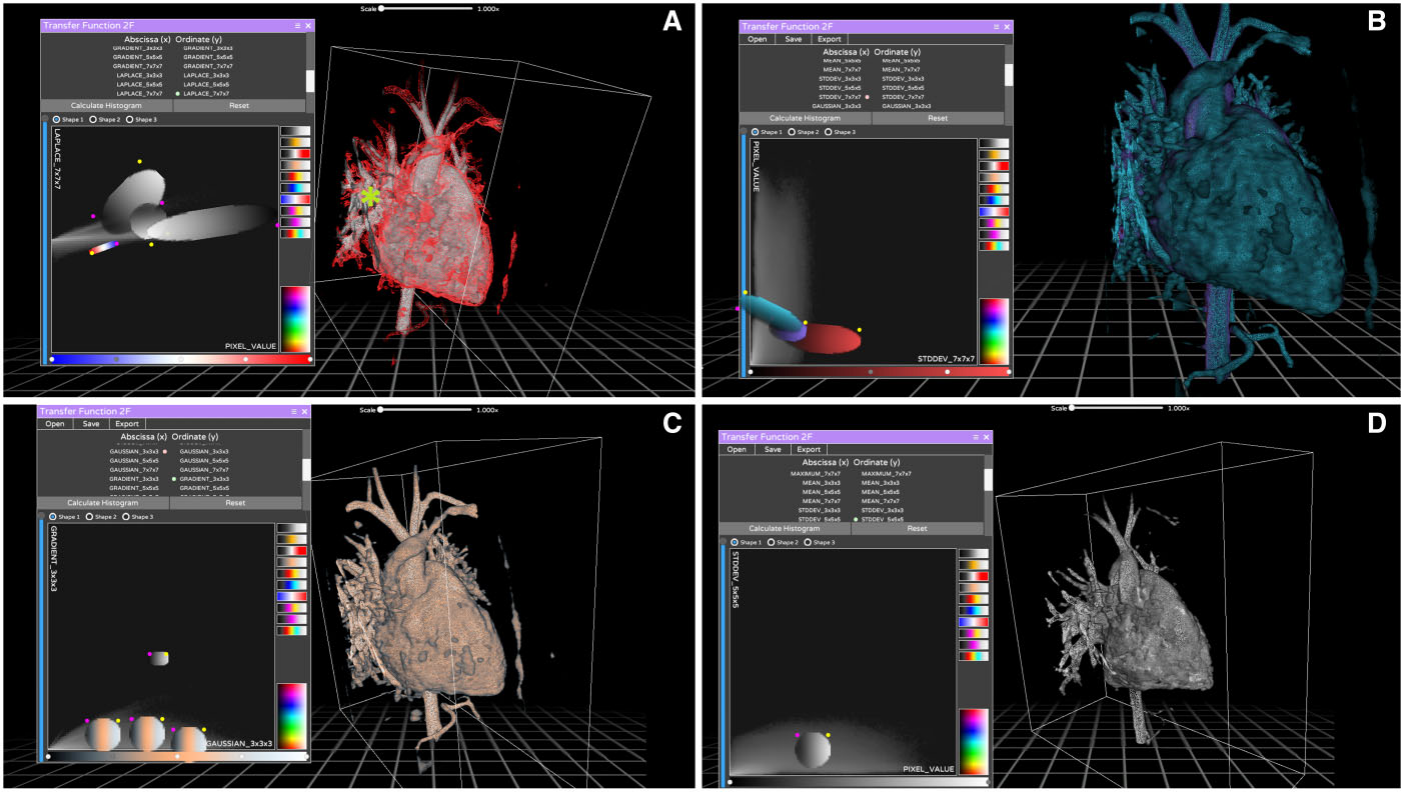
Reconstructions of case 4 generated by the participants. This patient had the worst quality examination in the series, so the reconstructions took longer to produce and were of poor quality overall. The diagnosis was partial anomalous pulmonary venous return of a single, large right superior pulmonary vein into the superior vena cava. A small pulmonary venous branch from the right middle lobedrained into the right atrium, and a small sinus venosus defect was present. Panel A shows the reference model created by the skilled user. Note how multiple filters had to be used, including a small red filter to enhance the outline of the model. However, the partial anomalous pulmonary venous return remains difficult to see due to the inherent low contrast images. Panel B shows a good result, with more monochrome filters. A small green star shows the defect itself. Panel C shows a good reconstruction, albeit with a single small unused filter. Panel D shows a complete lack of colour due to the use of a single greyscale filter.

The data were then evaluated, and the results of the expert user were compared with those of the 5 participants (Figs. 1–3). The quality of the reconstructions was rated by the senior author on a numerical scale from 0 to 5, where 0 was judged an unusable reconstruction and 5 a reconstruction that was comparable to or a better-quality model than the reference reconstruction. Other parameters considered when assigning this score were the use of colour to improve the morphological features, the visibility of all anatomical structures, the signal-to-noise ratio and the presence of artifacts. The analysis performed by the senior author was qualitative by design, so the score was based entirely on his judgement. That said, to make the process as objective as possible, a few parameters were used as landmarks. To be more precise, appropriate use of colour was rewarded with 1 point; good visibility of the anatomical defect was rewarded with 2 points; excessive presence of artifacts, either by insufficient cropping or suboptimal thresholds, was penalized by removing 1 point; insufficient contrast between the structures of interest and the other structures was also penalized by removing 1 point. We believe that, at a minimum, to be useful a 3D rendering needs to include the region of interest where the defect is located; it needs to be hollow to allow for internal exploration; and the main structures belonging to the heart need enough definition to be easily recognizable. Some models did not match these minimum requirements and were assigned a quality score of 1 to 2. The time required (in seconds) to perform the reconstruction was also recorded and compared among the participants. We then calculated a time score as the ratio between the time of the user and that of the expert multiplied by 5, thus obtaining an index between 0 and 5. The score was capped at 5; thus if the user was faster than the expert, this performance was automatically assigned a time score of 5. To balance performance and compensate for fast but imprecise or slow but skilled users, we then calculated an adjusted score derived from the arithmetic average between the time score and the quality score as an overall measure for a user result.

### Image acquisition and 3-dimensional reconstruction

Cardiac MRI was performed using a Philips Achieva 1.5 T scanner (Philips Medical Systems, Philips Healthcare, Amsterdam, the Netherlands). Images were acquired with a 5-channel phased-array heart coil. The 3D SSFP sequence, electrocardiogram and gated navigator in free breathing were performed for whole heart acquisition.

3D reconstructed models of the patients' anatomies were generated using DIVA software. This software was developed using the popular Unity game engine and includes 2 native interfaces: a desktop mode for volumetric viewing on a standard computer monitor (Video 1) and a VR mode for the interaction with medical images in an artificial environment through the use of a VR headset. Patient imaging data were loaded directly from Digital Imaging and Communications in Medicine (DICOM) files, and MRI volumetric renderings were generated instantly by the software, without any human intervention.

### Software and reconstruction pipeline description

Three different reconstruction tools were available in the software, namely, Maximum Intensity Projection, AVATAR Filter (a threshold drawn on the intensity histogram using different curves) and 2F transfer function (a complex tool that allows you to interact with bi-dimensional representations of the pixel space). Users were encouraged to experiment with them and find which one gave the best results. In particular, for the 2F transfer function, multiple combinations of X and Y axes were available to plot the pixel space in a 2-dimensional matrix of grey values, but very little explanation was provided on the exact meaning of each option. This was done on purpose, because a thorough understanding of those tools requires a mathematical and engineering background that is simply not available to most surgeons and doctors. Our goal was to demonstrate the feasibility of quick and easy training for naive medical staff; therefore no in-depth technical discussion was deemed necessary. Most users chose the 2F transfer feature as their preferred tool, but this was not subjected to judgment (Figs. 1–3).

The reconstruction pipeline starts with a simple folder containing the raw, unprocessed, DICOM images. After importation into the software, a Maximum Intensity Projection rendering is automatically generated, and the user has the option to crop the model in all 3 dimensions to isolate the region of interest as much as possible. Because a 3D rendering is already present, all following steps are optional. Finally, the user can pick among the tools available as well as customize the tools themselves to find the best combination of parameters to achieve the optimal reconstruction using a trial-and-error approach.

### Statistical analyses

All the scores were converted into a five-point Likert scale. Due to normal distribution, numerical variables were presented as mean ± standard deviation. A one-way repeated-measures analysis-of-variance (ANOVA) was used to compare the different cases. The *P*-value for statistical significance was set at 0.05. When a significant test result was obtained, a Bonferroni-adjusted post hoc analysis was used to assess pairwise differences between groups.

## RESULTS

### Case descriptions

*Case 1*: An 8-year-old female with PAPVR of the right superior pulmonary vein into the superior vena cava (SVC) well above the origin of the azygos vein. The right middle pulmonary vein drained into the junction between the SVC and the right atrium (RA). This defect was associated with a rather large sinus venosus defect (Fig. 1).
*Case 2*: A 31-year-old female with PAPVR of 2 separate right upper pulmonary veins in the SVC. The right middle pulmonary vein drained into the junction between the SVC and the RA, where there was a small sinus venosus defect (Fig. 2).
*Case 3*: A 26-year-old female with PAPVR of a single separated right superior pulmonary vein in the SVC near the origin of the azygos vein. The other pulmonary veins were draining normally, and a sinus venosus defect was also present.
*Case 4*: A 10-year-old boy with PAPVR of a single, large right superior pulmonary vein into the SVC. A small pulmonary venous branch from the right middle lobe drained into the RA and a small sinus venosus defect was present (Fig. 3).
Case 5: A 4-year-old girl with PAPVR of 2 right superior pulmonary veins draining into the SVC and 2 right middle pulmonary veins at the SVC-RA junction. One of the upper veins drained directly into the azygos vein.

### Scores obtained

The total scores obtained by the participants in case 1 were: quality score 2.4 ± 0.7; time 651 ± 280 s; time score 1.8 ± 0.9; adjusted score 2.1 ± 0.4. The total scores obtained by the participants in case 2 were: quality score 3.0 ± 1.4; time 402 ± 136 s; time score 1.5 ± 0.7; adjusted score 2.3 ± 0.5. The total scores obtained by the participants in case 3 were: quality score 3.2 ± 0.8; time 478 ± 165 s; time score 2.5 ± 1.1; adjusted score 2.8 ± 0.6. The total scores obtained by the participants in case 4 were: quality score 2.6 ± 1.1; time 345 ± 140 s; time score 3.8 ± 1.1; adjusted score 3.1 ± 0.5. The total scores obtained by the participants in case 5 were: quality score 4.2 ± 0.4; time 382 ± 62 s; time score 3.3 ± 0.5; adjusted score 3.8 ± 0.1. All scores and their averages are available in Table 1. Table 2 shows the same data organized by columns, as this may help clarify the fact that the study subjects are the participants.

**Table 1:** Scores obtained by the participants for 3-dimensional reconstruction with the DIVA software of each case of partial anomalous pulmonary venous return

User	Quality score	Time (s)	Time score	Adjusted score
**CASE 1**				
Expert	4	203	5	4.5
#1	1	312	3.3	2.2
#2	3	480	2.1	2.6
#3	2	894	1.1	1.6
#4	3	980	1	2.0
#5	3	589	1.7	2.4
**Total**	**2.4 ± 0.9**	**651 ± 281**	**1.8 ± 0.9**	**2.1 ± 0.4**
**CASE 2**				
Expert	5	109	5	5.0
#1	2	212	2.6	2.3
#2	2	310	1.8	1.9
#3	4	510	1.1	2.6
#4	5	520	1	3.0
#5	2	460	1.2	1.6
**Total**	**3.0 ± 1.4**	**402 ± 136**	**1.5 ± 0.7**	**2.3 ± 0.5**
**CASE 3**				
Expert	5	209	5	5.0
#1	3	241	4.3	3.7
#2	2	410	2.5	2.3
#3	3	660	1.6	2.3
#4	4	600	1.7	2.9
#5	4	480	2.2	3.1
**Total**	**3.2 ± 0.8**	**478 ± 165**	**2.5 ± 1.1**	**2.8 ± 0.6**
**CASE 4**				
Expert	4	249	5	4.5
#1	1	126	5	3.0
#2	3	300	4.2	3.6
#3	2	441	2.8	2.4
#4	3	480	2.6	2.8
#5	4	380	3.3	3.7
**Total**	**2.6 ± 1.1**	**345 ± 140**	**3.6 ± 1**	**3.1 ± 0.5**
**CASE 5**				
Expert	5	249	5	5.0
#1	4	326	3.8	3.9
#2	4	400	3.1	3.6
#3	4	371	3.4	3.7
#4	5	480	2.6	3.8
#5	4	336	3.7	3.9
**Total**	**4.2 ± 0.4**	**383 ± 62**	**3.3 ± 0.5**	**3.8 ± 0.1**

**Table 2:** Score averages obtained by the participants for 3-dimensional reconstruction with the DIVA software of each case of partial anomalous pulmonary venous return

Score averages	CASE 1	CASE 2	CASE 3	CASE 4	CASE 5
Quality score average	2.4 ± 0.9	3.0 ± 1.4	3.2 ± 0.8	2.6 ± 1.1	4.2 ± 0.4
Time average (s)	651 ± 280	402 ± 136	478 ± 165	345 ± 140	382 ± 62
Time score average	1.8 ± 0.9	1.5 ± 0.7	2.5 ± 1.1	3.8 ± 1.1	3.3 ± 0.5
**Adjusted score average**	**2.1 ± 0.4**	**2.3 ± 0.5**	**2.8 ± 0.6**	**3.1 ± 0.5**	**3.8 ± 0.1**

The variation of the scores over time was significant for all the parameters analysed: quality score (*P* = 0.028), time (*P* < 0.001), time score (*P* < 0.001), adjusted score (*P* < 0.001).

## DISCUSSION

The last 50 years have been characterized by a marked improvement in the diagnosis and treatment of various CHD. A crucial role in the management and correction of this broad spectrum of disease is represented by the understanding of the anatomy and physiology of each case. For this reason, several studies are usually performed in the preoperative period, including CT scans, echocardiography and MRI. With this technological development, it is now possible to obtain much more detailed and accurate preoperative details. Nevertheless, the surgeon can still run into unexpected anatomical problems that can negatively affect the final outcome.

3D technologies initially emerged as a resource for teaching purposes. However, they probably have reached their maximum utility in preoperative surgical planning. As a matter of fact, their use reduces operative times and can completely change the planned surgery [2]. 3D cardiac reconstructions are created mainly from CT scans and less frequently from MRI or ultrasound images. One of the limiting elements in 3D reconstruction is the time taken to obtain a final Standard Tessellation Language (STL) file (which is the file required either for 3D printing or VR). Another element to consider is the need for a team with a high level of 3D expertise and who can use the software currently available. Therefore, this technology is currently limited and used only in some centres across Europe.

DIVA software is a new software that allows a fast-track reconstruction of cardiac images in 3D models. Indeed, the innovative and unique element of this software is the short time required to generate a reconstruction. In our previous studies, we demonstrated the potential use of DIVA in different CHD [4]. In this study we wanted to confirm our results with inexperienced users compared to an expert.

All our participants recreated 3D models in a relatively short time, maintaining a good overall quality (mean quality score always ≥ 3 on a 1–5 scale). The models were then uploaded to a VR environment. The accuracy and precision of the anatomical details were excellent. These features allow wider use of 3D technology even in small hospitals or centres without 3D modelling experts, to implement and improve potential surgical planning. The scores collected by users varied. However, the overall trend of all the parameters analysed showed a statistical improvement between case 1 and case 5. This result suggests that the learning curve is linear and that, although the results have improved gradually, the former were already of sufficient quality to be useful. This is particularly important in setting up accelerated model generation. Indeed, long reconstruction protocols that guarantee accuracy only after considerable experience would defeat the main purpose of this software, which is to allow “lay” users to reconstruct 3D DICOM images themselves. The main advantages of this technology lie in speed and realism, the latter mainly attributed to the immersive 3D experience that can be achieved through a VR headset. Despite the cost barrier, the required hardware is widely available and mass-produced as gaming peripherals, with the base system not even requiring a VR headset, thus allowing for gradual upgrades if needed. The cross-platform compatibility of the software is guaranteed by the use of the Unity engine. Our surgeons have found the VR experience to be superior to the 2D images offered by most 3D segmentation software, primarily due to the internal navigation and manipulation that become possible using a VR headset. This feature adds information and details about the anatomical conformation of internal structures without adding too much additional complexity, if any, to the user's experience.

Our selection of a simple extracardiac defect was intentional, and our goal was to create the optimal conditions for evaluation of software performance. However, we have also evaluated the effectiveness of this tool with more complex intracardiac defects, such as an atrioventricular septal defect or double outlet right ventricle, which presented a challenging set-up problem. The senior author (chief congenital heart surgeon at our institution) later reported that he found the tool useful, because it allowed for intracardiac navigation and familiarity with various proportions and structural orientations. However, quantifying such outcomes presents significant challenges, requiring a case series or, preferably, a clinical study capable of measuring improved clinical outcomes such as operating time and patient morbidity.

Finally, we made a conscious decision to limit our investigation to cardiac MRI only, to achieve maximum homogeneity of the data set. Although it is conceivable to use other imaging modalities such as CT scans, we have refrained from such practices to rule out potential bias. Using a heterogeneous data set could give an unfair advantage to similar cases, as demonstrated by the sample case used in the training video tutorial, which would have inevitably been captured using CT or MRI imaging.

## CONCLUSIONS

3D models and VR are becoming important technologies for preoperative assessment and surgical planning in the treatment of CHD. DIVA software is a very simple software that allows accurate 3D reconstruction in a relatively short time (“fast-track VR”). In this study, we have demonstrated the potential use of DIVA software by inexperienced users, with a significant improvement in quality and time after performing a few cases. Further studies are needed to confirm the potential application of this technology on a larger scale.

## Funding

The whole project was supported by general funding, thus unrelated to the specific topic addressed in our paper.

**Conflict of interest:** Mohamed El Beheiry and Jean-Baptiste Masson are cofounders, shareholders and, respectively, Chief Technology Officer and Chief Scientific Officer of AVATAR MEDICAL SAS, a start-up company that commercializes software for surgery planning in virtual reality. The DIVA software used in this study is not being commercialized by AVATAR MEDICAL SAS, although the company’s technology is based on the same technology. The DIVA software, which serves as base for this study, is freely available and has been reported previously in the literature [3]. All developments within this paper are open source. AVATAR MEDICAL played no role in the design or realization of this study.

## Data Availability

The data underlying this article will be shared upon reasonable request to the corresponding author.
